# Pancreatic tail splenunculus: Case report and review of the literature

**DOI:** 10.1016/j.ijscr.2019.02.020

**Published:** 2019-02-28

**Authors:** Ricky H. Bhogal, Andrew Wotherspoon, I. Zerizer, Aamir Z. Khan

**Affiliations:** a Department of Academic Surgery, The Royal Marsden Hospital, Fulham Road, Chelsea, London, SW3 6JJ, United Kingdom; b Department of Histopathology, The Royal Marsden Hospital, Fulham Road, Chelsea, London, SW3 6JJ, United Kingdom; c Department of Nuclear Medicine and Radiology, The Royal Marsden Hospital, Fulham Road, Chelsea, London, SW3 6JJ, United Kingdom

**Keywords:** Pancreas, Splenunculus, Neuroendocrine tumour, Nuclear scintigraphy

## Abstract

**Introduction:**

Mass lesions in the pancreatic tail are increasingly identified upon radiological imaging. However the diagnosis of these lesions can be challenging and impact significantly upon patient management.

**Presentation of case:**

We report a case of an intrapancreatic accessory spleen initially diagnosed as a neuroendocrine tumour of the pancreas tail following nuclear scintigraphy.

**Discussion:**

The investigation of solitary pancreatic tail lesions and the potential management paradigms are discussed.

**Conclusion:**

Solitary lesions within the pancreatic tail should have splenunculus included in the differential diagnosis.

## Highlights

• Solitary pancreatic tail lesion should undergo nuclear scintigraphy to assess whether the lesion is compatible with a splenunculus.

## 1 Case report

**Fig. 1:**
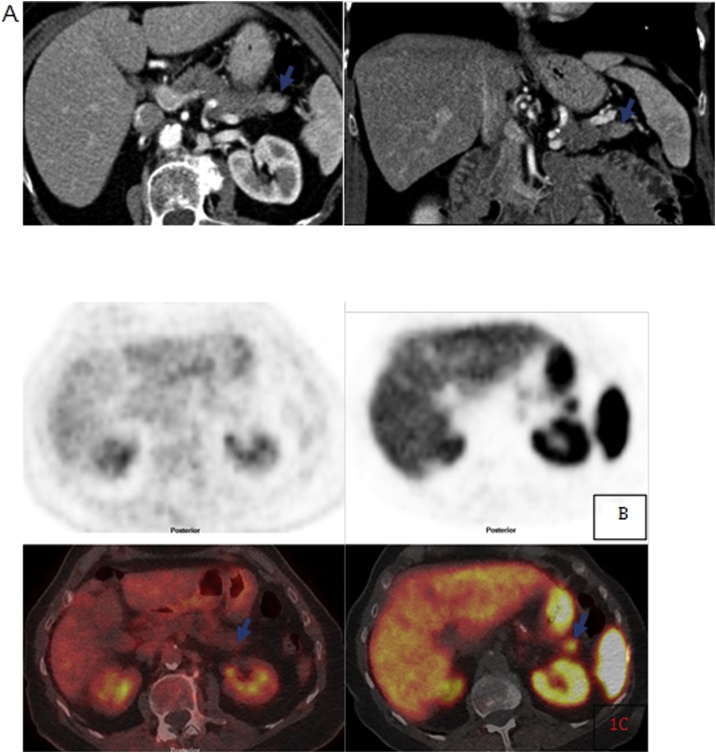
(A) Axial and coronal CT arterial phase images show an arterially enhancing intraparenchymal pancreatic tail lesion (arrows) (B) and (C) Axial PET and PET/CT images FDG (1b) show no uptake in the pancreatic tail lesion whilst the DOTATATE (1c) images show increased somatostatin receptor expression in the pancreatic tail lesion (arrows).

**Fig. 2:**
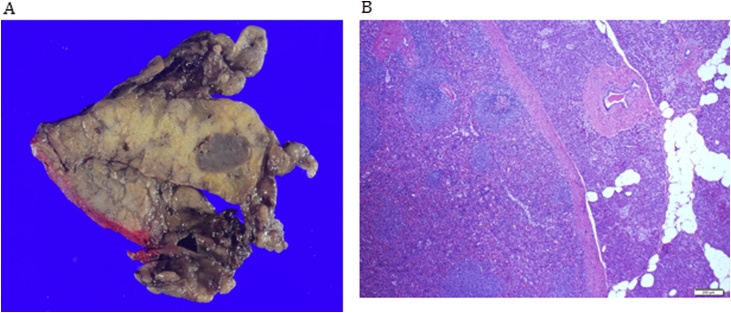
A: Macroscopic examination of the intra-pancreatic nodule revealed features resembling splenic tissue with the presence of Malpigian corpuscles within a vascular red pulp background. B: Histological examination showed encapsulated splenic tissue with normal red and white pulp.

The following case has been reported as per SCARE guidelines [1]. A 68 year-old female patient with a recent diagnosis of Barrett’s oesophagus and known moderate-severe chronic obstructive pulmonary disease (COPD) underwent endoscopic ultrasound (EUS) as part of oesophageal surveillance. The linear EUS demonstrated an incidental finding of a solid lesion of 12 × 7 mm within the tail of pancreas. Fine needle aspiration of the lesion was non-diagnostic. Subsequent Computed Tomography (CT) of the thorax, abdomen and pelvis demonstrated a hypervascular lesion within the pancreatic tail and an indeterminate left upper lung nodule (Fig. 1A). The pancreatic lesion appeared to arising from the pancreatic parenchyma with no fat plane separation. Both the pancreatic lesion and lung nodule demonstrated no fluorodeoxyglucose (FDG) avidity (Fig. 1B). The likely diagnosis was thought to be a non-FDG avid pancreatic malignancy such as a pancreatic neuroendocrine tumour (NET). Ga^68^ DOTATATE PET/CT demonstrated a somatostatin receptor positive small pancreatic lesion that consistent with a well differentiated NET (Fig. 1C). The lung nodule was somatostatin receptor negative and there was no other evidence of metastatic disease. In addition gut hormone profile and urinary 5-hydroxyindoleacetic acid were reported within normal limits. Following multi-disciplinary team discussion a recommendation of thorascopic resection of the lung nodule was made to exclude metastatic disease. Histopathological analysis of the lung nodule was consistent with a hyperplastic nodule but the lung resection was complicated by a pneumothorax requiring prolonged hospitalisation. Lung function tests prior to abdominal surgery revealed a forced expiratory volume in 1 s (FEV1) 48% of predicted, forced vital capacity (FVC) 80% of predicted and a FEV1/FVC ratio of 0.45. In addition VO2 peak was 12 ml/kg/min and anaerobic threshold (AT) 8.5 ml/kg/min. Although the patient was high risk for surgery given the possibility of pancreatic neoplasm after appropriate counselling the patient consented to laparoscopic distal pancreatectomy and splenectomy. The lesion was identified by intraoperative ultrasound and the pancreas proximal to the lesion stapled. Blood loss was 80mls and operating time 135 min. Abdominal drainage was removed on day 2 post-op and the patient discharged on day 7. Histological analysis revealed a splenunculus (accessory spleen) within the pancreatic tail (Fig. 2). The patient is well 6 months after surgery having made a full recovery.

## 2 Discussion

The spleen is formed from mesenchymal cells found between the layers of the dorsal mesogastrium. Additional splenic tissue or splenunculi can be acquired or congenital. In acquired cases it is generally believed to result from trauma with cells then taking a blood supply from surrounding structures [2]. Thus splenic tissue has been reported in the lung and liver following trauma [3,4].

Congenital cases of accessory splenic tissue are usually solitary nodules of functioning tissue and found in the region of the gastrosplenic ligament or spleno-pancreatic ligaments. These nodules are usually incidentalomas and as such are asymptomatic but need to be differentiated from neoplastic lesions. Indeed 16% of abdominal CTs and up to 30% of autopsies report splenunculi [5] with 80% of accessory spleens being at the splenic hilum although not related to the pancreatic tail [6].

On CT imaging splenunculi can be very challenging, typically these are a few centimeters in diameter well-circumscribed ovoid nodules that have similar density and enhancement characteristics to the native spleen but are seen separate to it. Furthermore splenunculi which are intraparenchymal can mimic NETs as they have similar appearances on CT and magnetic resonance imaging [7]. The hypervascular lesion, as reported in our patient, raised the possibility of a neoplasm such as NETs. Moreover, the lesion on CT appeared to be arising from the pancreatic parenchyma on both axial and coronal imaging with no clear fat plane separation. In addition, the Ga^68^ DOTATATE PET CT demonstrated avidity of the pancreatic tail lesion and hence the lesion was thought to be a NET. However, the spleen has a high density of somatostatin receptors and shows intense uptake on somatostatin receptor imaging, therefore spleunculi can give rise to false positive results [8,9]. Given the high risk nature of our patient, a Technetium-^99^m heat-damaged red blood cell or Technetium-^99^m sulfur colloid scans would have demonstrated positive uptake with a splenunculus but not with a NETs allowing a conservative approach in the management of our patient [8,9]. The authors would like to highlight this pitfall and increase awareness of this entity to avoid unnecessary surgery.

In summary, solitary nodules within the pancreatic tail have a differential diagnosis which includes benign and malignant entities and full investigations including nuclear medicine studies to exclude splenunculi should be undertaken prior to definitive management decisions.

## Conflicts of interest

No conflict of interests to declare for all authors.

## Sources of funding

Not applicable.

## Ethical approval

This case report does not require ethical approval for publication by our institution (The Royal Marsden Hospital). It is satisfactory that the patient has consented for publication of the report.

## Consent

Written informed consent was obtained from the patient for publication of this case report and accompanying images. A copy of the written consent is available for review by the Editor-in-Chief of this journal on request.

## Author contribution

RHB – writing of the paper, literature review and critical appraisal

AW – data analysis

IZ – data analysis

AZK – data analysis and writing of the paper.

## Registration of research studies

Not applicable.

## Guarantor

RHB.

## Provenance and peer review

Not commissioned, externally peer-reviewed.
